# Minimal Clinically Important Differences in Conservative Treatment Versus Hip Arthroscopy for Femoroacetabular Impingement Syndrome: A Frequentist Meta‐Analysis of RCTs

**DOI:** 10.1111/os.70097

**Published:** 2025-07-05

**Authors:** Nikolai Ramadanov, Jonathan Lettner, Maximilan Voss, Robert Prill, Robert Hable, Dobromir Dimitrov, Roland Becker

**Affiliations:** ^1^ Center of Orthopaedics and Traumatology Brandenburg Medical School, University Hospital Brandenburg an der Havel Brandenburg an der Havel Germany; ^2^ Faculty of Health Science Brandenburg Brandenburg Medical School Theodor Fontane Brandenburg an der Havel Germany; ^3^ Faculty of Applied Computer Science Deggendorf Institute of Technology Deggendorf Germany; ^4^ Department of Surgical Propedeutics Faculty of Medicine, Medical University of Pleven Pleven Bulgaria

**Keywords:** femoroacetabular impingement, hip arthroscopy, meta‐analysis, systematic review

## Abstract

Several meta‐analyses of surgical versus non‐operative treatment of femoroacetabular impingement syndrome (FAIS) have been published, but reliable evidence is still lacking. The aim of this meta‐analysis of randomized controlled trials (RCTs) was to assess the outcomes of FAIS patients treated conservatively compared with those treated with hip arthroscopy (HAS). PubMed, CENTRAL of the Cochrane Library, Epistemonikos, and Embase databases were searched up to March 31, 2025. Quality was assessed using the Cochrane Risk of Bias 2 tool, the level of evidence for each outcome parameter was determined using the GRADE system, and publication bias was presented in funnel plots. In a common effect and random effects meta‐analysis, mean differences (MDs) between the conservative treatment group and the HAS group were calculated with 95% confidence intervals (CIs) using the Hartung‐Knapp‐Sidik‐Jonkman heterogeneity estimator. A total of 7 RCTs with a total of 489 patients in the conservative treatment group and 484 patients in the HAS group met the inclusion criteria. Of the 7 RCTs included, four were assessed as having a low risk of bias, one as having a moderate risk of bias, and two as having a high risk of bias. The outcomes “post‐intervention functional MCID” and “iHOT at ≤ 12 months post‐intervention” had a high level of evidence, and the outcome “HOS‐ADL at ≤ 8 months post‐intervention” had a moderate level of evidence. No significant publication bias was detected for any outcome. The HAS group had a statistically significant 0.85 higher post‐intervention functional MCID (common effect model: MD: 0.85 CIs 0.53–1.17; random effects model: MD: 0.85 CIs 0.64–1.06; *I* ^2^ = 0%; *τ* ^2^ = 0.02; *p* = 0.96) and a statistically significant 10.74 higher iHOT at ≤ 12 months post‐intervention than the conservative treatment group (common effect model: MD: 10.74 CIs 7.06 to 14.42; random effects model: MD: 10.98 CIs 6.62 to 15.34; *I* ^2^ = 0%; *τ* ^2^ = 7.52; *p* = 0.62). There was no difference between the HAS group and the conservative treatment group in HOS‐ADL at ≤ 8 months post‐intervention (common effect model: MD: 5.62 CIs 1.76 to 9.48; random effects model: MD: 4.10 CIs −12.31 to 20.50; *I* ^2^ = 69%; *τ* ^2^ = 29.88; *p* = 0.04). This meta‐analysis using high‐quality statistical methods showed a statistically significant higher post‐intervention functional MCID and iHOT at ≤ 12 months post‐intervention in favor of the HAS group compared to the conservative treatment group. HOS‐ADL at ≤ 8 months post‐intervention showed no differences.

## Graphical abstract

This meta‐analysis of 7 RCTs (973 patients) compared hip arthroscopy (HAS) with conservative treatment for femoroacetabular impingement syndrome. HAS showed significantly higher post‐intervention functional MCID (+0.85) and iHOT at ≤ 12 months (+10.74). No differences were found in HOS‐ADL at ≤ 8 months. Statistical methods ensured high evidence reliability.

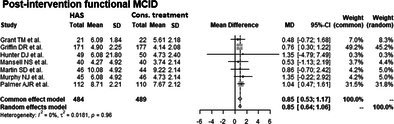

**Abbreviations**
CI: confidence interval
FAIS: femoroacetabular impingement syndrome
GRADE: Grading of Recommendations Assessment, Development and Evaluation
HAS: hip arthroscopy
HHS: Harris Hip Score
HK: Hartung‐Knapp
HOOS: Hip disability and Osteoarthritis Outcome Score
HOS‐ADL: Hip Outcome Score—Activities of Daily Living
iHOT: International Hip Outcome Tool
MCID: minimal clinically important difference
NRS: Numeric Rating Scale
PRISMA: Preferred Reporting Items for Systematic Reviews and Meta‐Analyses
PROM: patient‐reported outcome measure
PROSPERO: International Prospective Register of Systematic Reviews
RCT: randomized controlled trial
RoB: risk of bias
VAS: Visual Analog Scale

## 1 Introduction

In recent decades, the understanding of the etiology, pathogenesis, and consequences of femoroacetabular impingement syndrome (FAIS) for the hip joint has improved considerably. This has led to an increase in related publications. The higher number of primary studies has led to a total of 11 meta‐analyses [1, 2, 3, 4, 5, 6, 7, 8, 9, 10, 11] being published between 2020 and 2025, comparing the outcomes of conservatively treated FAIS patients with those of hip arthroscopy (HAS). Collectively, these meta‐analyses [1, 2, 3, 4, 5, 6, 7, 8, 9, 10, 11] included 23 different primary studies [12, 13, 14, 15, 16, 17, 18, 19, 20, 21, 22, 23, 24, 25, 26, 27, 28, 29, 30, 31, 32, 33, 34]. However, some of these meta‐analyses have serious methodological flaws (Tables 1 and 2). One [6] of the 11 meta‐analyses [1, 2, 3, 4, 5, 6, 7, 8, 9, 10, 11] was retracted [35] and subsequently republished [7], with revisions in the interpretation of results and the conclusions. Most of these meta‐analyses [1, 2, 3, 4, 5, 6, 7, 8, 10, 11] did not specify the heterogeneity estimator used, nor do they appear to have used a Hartung‐Knapp (HK) adjustment. A recently published epidemiological study [36] showed that using a weak heterogeneity estimator without HK adjustment in hip replacement meta‐analyses can lead to spurious results. Another shortcoming of these meta‐analyses [1, 2, 3, 4, 5, 6, 7, 8, 10, 11] is that they did not adequately consider the role of the minimal clinically important difference (MCID) in interpreting the clinical relevance of functional outcome parameters. It is particularly striking that of these 11 meta‐analyses [1, 2, 3, 4, 5, 6, 7, 8, 9, 10, 11], 6 [1, 2, 3, 4, 5, 10] included only 3 primary studies [13, 15, 18], which were also completely identical, but somehow these 6 meta‐analyses produced different results [1, 2, 3, 4, 5, 10]. Saueressig et al. performed a critical analysis of these 6 meta‐analyses on FAIS and recalculated them using appropriate methods [37]. In conclusion, these six meta‐analyses [1, 2, 3, 4, 5, 10] were shown to have unreliable results due to methodological flaws [37]. Our recently published multilevel meta‐analysis [9] of 21 RCTs [12, 13, 14, 15, 16, 17, 18, 20, 21, 22, 23, 24, 25, 26, 27, 28, 29, 30, 31, 32, 33] evaluated the HAS and conservative treatment subgroups using a comparison of subgroup means. However, this meta‐analytical approach requires validation through a direct comparison using a traditional frequentist meta‐analysis that calculates mean differences between the two treatment groups. There is currently insufficient evidence that HAS is superior to physiotherapy for FAIS. There is a need for methodologically sound meta‐analyses to fill this gap in orthopedic research.

**TABLE 1:** Comparison of the published meta‐analyses on treatment of FAIS.

Meta‐analyses	Year of publication	Primary studies included	Patients, *N*	Heterogeneity estimator	Statistical model	Outcome	Statistically significant result	Reaching MCID
Bastos RM et al. [1]	2021	3 [13, 15, 18]	650	NR	REM	iHOT, HOS	No difference	No
Casartelli NC et al. [2]	2021	3 [13, 15, 18]	650	NR	REM	iHOT	HAS better	Yes
Dwyer T et al. [3]	2020	3 [13, 15, 18]	650	NR	FEM	iHOT	HAS better	No
Ferreira GE et al. [4]	2021	3 [13, 15, 18]	650	NR	REM	QoL	HAS better	Yes
Gatz M et al. [5]	2020	3 [13, 15, 18]	644	NR	FEM/REM	HOS, iHOT	HAS better	Yes
Lamo‐Espinosa JM et al. [6]	2023	Retracted article [35]						
Lamo‐Espinosa JM et al. [7]	2025	6 [13, 14, 15, 16, 18, 19]	839	NR	FEM/REM	iHOT, HOS	HAS better	No
Mahmoud SSS et al. [8]	2022	4 [13, 14, 15, 18]	749	NR	FEM/REM	iHOT	HAS better	No
Ramadanov N et al. [9]	2025	21 [12, 13, 14, 15, 16, 17, 18, 20, 21, 22, 23, 24, 25, 26, 27, 28, 29, 30, 31, 32, 33]	1799	REML with HK adjustment	REM	HHS, iHOT, HOOS, HOS, NRS, VAS	HAS better	No
Schwabe MT et al. [10]	2020	3 [13, 15, 18]	650	NR	REM	iHOT, HOS	HAS better	Yes
Zhu Y et al. [11]	2022	6 [13, 14, 15, 16, 18, 34]	1187	NR	FEM/REM	HOS, iHOT	HAS better	Yes

Abbreviations: FAIS, femoroacetabular impingement syndrome; FEM, fixed effects model; HAS, hip arthroscopy; HOS, Hip Outcome Score; HHS, Harris Hip Scores; HK, Hartung‐Knapp; HOOS, Hip disability and Osteoarthritis Outcome Score; iHOT, International Hip Outcome Tool; NRS, Numeric Rating Scale; QoL, quality of life; REM, random effects model; REML, restricted maximum likelihood; VAS, Visual Analog Scale.

**TABLE 2:** Meta‐analyses quality assessment, using AMSTAR 2.

Author	Protocol registered before commencement of the review	Adequacy of the literature search	Justification for excluding individual studies	RoB from individual studies being included in the review	Appropriateness of meta‐analytical methods	Consideration of RoB when interpreting the results of the review	Assessment of presence and likely impact of publication Bias	Overall Quality
Bastos RM et al. [1]	High	High	High	High	Low	Low	Critically low	Low
Casartelli NC et al. [2]	High	High	Moderate	High	Low	Low	High	Moderate
Dwyer T et al. [3]	Critically low	High	Moderate	High	Low	Low	Critically low	Critically low
Ferreira GE et al. [4]	High	High	High	High	Low	Low	Critically low	Low
Gatz M et al. [5]	Critically low	High	Moderate	High	Low	Low	High	Low
Lamo‐Espinosa JM et al. [7]	High	High	Moderate	High	Low	Low	High	Moderate
Mahmoud SSS et al. [8]	High	High	Moderate	High	Low	Low	Critically low	Low
Ramadanov N et al. [9]	High	High	High	High	High	Low	Moderate	Moderate
Schwabe MT et al. [10]	Critically low	High	Moderate	High	Low	Low	Critically low	Critically low
Zhu Y et al. [11]	Critically low	High	Moderate	High	Low	Low	Critically low	Criticlly low

*Note*: Possible scoring results: “critically low,” “low,” “moderate,” and “high.” The labels refer to quality and not to RoB, for example, “low” is poor and “high” is good. The meaning of “low” and “high” is reversed in the RoB 2 tool compared to AMSTAR 2.

Abbreviations: AMSTAR 2, A MeaSurement Tool to Assess Systematic Reviews 2; RoB, risk of bias.

The aim of this meta‐analysis of RCTs was, in a direct comparison, to evaluate the outcomes of FAIS patients treated conservatively compared with those treated with HAS.

## 2 Methods

### 2.1 Data Sources and Search Strategies

The study protocol was registered in the International Prospective Register of Systematic Reviews (PROSPERO) on June 6, 2024 (CRD42024551224). The previously published multilevel meta‐analysis [9] was also conducted under the same protocol but followed an alternative statistical approach and included a broader set of primary studies [12, 13, 14, 15, 16, 17, 18, 20, 21, 22, 23, 24, 25, 26, 27, 28, 29, 30, 31, 32, 33]. We followed the updated PRISMA guidelines [38] to maintain the highest scientific standards. The PRISMA checklist is available in the Supplementary Appendix (Data S1). PubMed, CENTRAL of the Cochrane Library, Epistemonikos, and Embase were searched for RCTs comparing conservative treatment and HAS in FAIS patients up to March 31, 2025. A BOOLEAN search strategy was tailored to the syntax of each database: (((femoroacetabular impingement) OR (FAI)) AND ((arthroscopy) OR (conservative) OR (physiotherapy))). No restrictions on year of publication or language were applied.

### 2.2 Study Selection and Inclusion/Exclusion Criteria

Two independent reviewers (Nikolai Ramadanov and Jonathan Lettner) assessed the titles and abstracts of the identified records. Relevant records were further assessed by full‐text analysis. The final decision to include each study was made by consensus between the two reviewers (Nikolai Ramadanov and Jonathan Lettner). The kappa coefficient (*κ*) was used to assess the agreement between the reviewers.

The inclusion criteria for this meta‐analysis included only RCTs comparing conservative treatment and HAS in FAIS patients. Conservative treatment included non‐surgical approaches such as physiotherapy, pharmacological pain relief, and lifestyle changes.

Trials were excluded for the following reasons: (i) did not report an outcome of interest; (ii) reported incomplete outcome data that confounded the result; (iii) lack of randomization; (iv) RCTs comparing two different conservative treatment groups or two different HAS groups.

### 2.3 Data Extraction and Outcome Measures

Data extraction was performed by two independent reviewers (Jonathan Lettner and Maximilan Voss). Disagreements were resolved by discussion and consensus with a third reviewer (Nikolai Ramadanov). Various information such as first author, year of publication, study origin, number of patients included, patient characteristics, study design, risk of bias, relevant outcome parameters, and duration of follow‐up were extracted. These data were then exported from the text of the included RCTs into an Excel spreadsheet (see Data S2) for appropriate statistical analysis.

Hip function and quality of life were measured using several patient‐reported outcome measures (PROMs), including the Harris Hip Score (HHS) to assess hip function and pain, the International Hip Outcome Tool (iHOT) to assess hip‐related quality of life, and the Hip Outcome Score—Activities of Daily Living (HOS‐ADL) to assess hip function in activities of daily living. Where PROMs were reported as subscales, they were combined into a total score. Conversion to MCID units [39] was performed by dividing the PROM score by the most conservative MCID reported in the literature [40, 41]. Nwachukwu et al. [40] found a MCID of 8.20 for the HHS and a MCID of 12.00 for the iHOT. Kemp et al. [41] found a MCID of 9.00 for the HOS‐ADL. In RCTs where multiple PROMs were reported, we selected the PROMs in the following order: HHS, iHOT, and HOS‐ADL.

### 2.4 Quality Assessment

Two independent reviewers (Jonathan Lettner and Maximilan Voss) individually assessed the quality of the RCTs included in the analysis. Risk of bias (RoB) was assessed using the Cochrane RoB 2 tool [42], which provides a comprehensive assessment framework. The level of evidence for each outcome parameter was determined according to the established criteria of the GRADE system [43], which allows a thorough analysis of the quality of the evidence. Disagreements between the reviewers (Jonathan Lettner and Maximilan Voss) were resolved by consensus. In addition, publication bias was assessed and presented in funnel plots.

### 2.5 Measures of Treatment Effect

Statistics were performed by a professional statistician (Robert Hable) using the R packages meta and metafor. Both common effect and random effects models were tested because heterogeneity was low to high for some outcome parameters. Therefore, in frequentist meta‐analysis mean differences (MDs) with 95% CIs were calculated for continuous outcomes using the Sidik‐Jonkman heterogeneity estimator with HK [44] adjustment. Study weighting was performed using inverse variance. Statistical heterogeneity was assessed using the Higgins *I* ^2^ test, which categorizes heterogeneity as low (< 25%), moderate (25%–75%) or high (> 75%) [45]. The *p*‐value from Cochran's *Q*‐test was used to assess heterogeneity, with a *p*‐value below 0.05 indicating statistical significance. Forest plots were used to graphically present the results of individual studies and the respective pooled effect size estimate.

## 3 Results

### 3.1 Systematic Review

**FIGURE 1:**
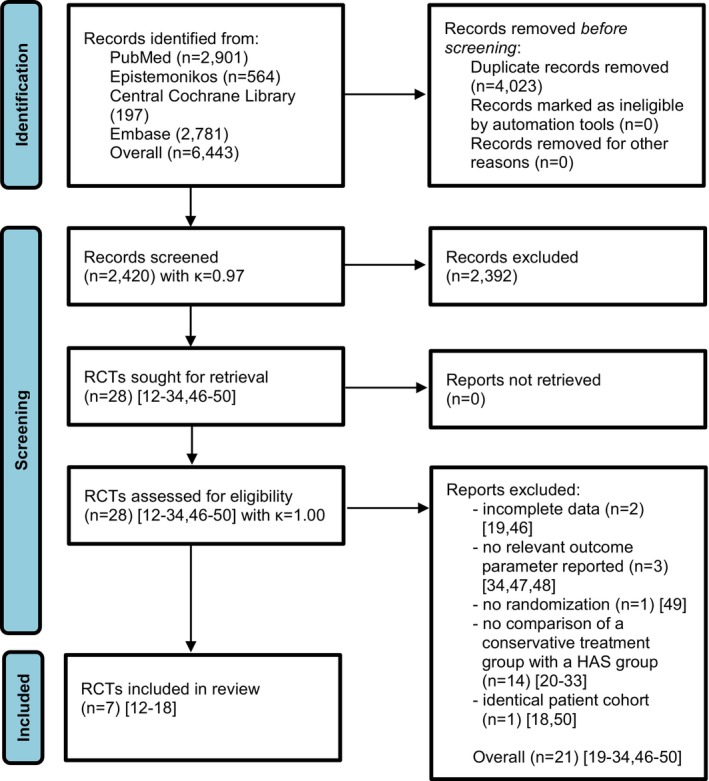
PRISMA flow diagram of the search results and selection according to our inclusion criteria. HAS, hip arthroscopy; RCT, randomized controlled trial.

In the systematic review of PubMed, CENTRAL, Epistemonikos, and Embase, a total of 2420 records were screened for title and abstract with high inter‐reviewer agreement (*κ* = 0.97) after the removal of 4023 duplicates. A total of 28 RCTs [12, 13, 14, 15, 16, 17, 18, 19, 20, 21, 22, 23, 24, 25, 26, 27, 28, 29, 30, 31, 32, 33, 34, 46, 47, 48, 49, 50] were assessed for eligibility with full inter‐reviewer agreement (*κ* = 1.0) whereas 2 RCTs were excluded because they provided incomplete outcome data [19, 46], 3 RCTs were excluded because they did not report an outcome of interest [34, 47, 48], 1 study had no randomization [49], and 14 RCTs were excluded because they did not compare a conservative treatment group with a HAS group, but two different conservative treatment groups or two different HAS groups with themselves [20, 21, 22, 23, 24, 25, 26, 27, 28, 29, 30, 31, 32, 33]. Finally, another RCT [50] was a mid‐term follow‐up of an already included RCT [18], both RCTs sharing the same patient cohort. The systematic review of the literature identified 7 RCTs [12, 13, 14, 15, 16, 17, 18] with a total of 973 patients that met the eligibility criteria for inclusion in the meta‐analysis (Figure 1).

### 3.2 Characteristics of the Included RCTs

The main characteristics of the seven included RCTs are shown in Table 3. A total of 489 patients were included in the conservative treatment group and 484 patients in the HAS group. The mean age of the conservative treatment group was 35.8 years (range: 30.6–49.1), 59.1% of the patients were men, and the BMI averaged 25.8 kg/m^2^ (range: 24.1–27.5). In the conservative treatment group, the duration of pain averaged 936.0 days (range: 548.0–1217.0) and the α angle averaged 69.0 (range: 64.0–70.7). In the HAS group, the mean age was 36.1 years (range: 29.7–49.6), 51.9% of the patients were men, and the body mass index (BMI) averaged 25.8 kg/m^2^ (range: 23.5–28.2). In the HAS group, the duration of pain averaged 848.7 days (range: 691.0–1125.0) and the α angle averaged 67.8 (range: 61.0–70.3).

**TABLE 3:** Main characteristics of the RCTs included.

Author	Year of publication	Origin	Treatment	Patients, *N*	Male sex, *N* (%)	FAIS type, *N* (%)	Age (years), SD; range	BMI (kg/m^2^), SD; range	Duration of pain (days), range	α angle (degree), SD; range	Follow‐up (months)	Extracted PROM for post‐intervention functional MCID calculation
Grant et al. [12]	2022	Australia	Cons.	22	13 (59.1%)	Cam: 17 (77.3%)	34.0 ± 9.5	24.1 ± 2.3	NR	70.5 ± 16.0	12	iHOT at 12 months post‐intervention
						Pincer: 2 (9.1%)						
						Mixed: 3 (13.6%)						
			HAS	21	11 (52.4%)	Cam: 14 (66.7%)	34.6 ± 11.4	23.5 ± 3.2	NR	69.8 ± 8.7		
						Pincer: 2 (9.5%)						
						Mixed: 5 (23.8%)						
Griffin et al. [13]	2018	UK	Cons.	177	113 (63.8%)	Cam: 133 (75.1%)	35.2 ± 9.4	NR	1217; 243–1217	64.0 ± 18.0	12	iHOT at 6 months post‐intervention
						Pincer: 14 (7.9%)						
						Mixed: 30 (17.0%)						
			HAS	171	100 (58.5%)	Cam: 129 (75.4%)	35.4 ± 9.7	NR	1125; 183–1095	61.0 ± 17.0		
						Pincer: 13 (7.6%)						
						Mixed: 29 (17.0%)						
Hunter et al. [14]	2021	Australia	Cons.	50	26 (52.0%)	Cam: 32 (64.0%)	32.9 ± 9.1	NR	548; 76–3650	70.6 ± 15.6	12	iHOT at 6 months post‐intervention
						Pincer: 9 (18.0%)						
						Mixed: 9 (18.0%)						
			HAS	49	31 (63.3%)	Cam: 30 (61.2%)	32.9 ± 11.8	NR	730; 61–2555	70.2 ± 11.9		
						Pincer: 9 (18.4%)						
						Mixed: 10 (20.4%)						
Mansell et al. [15]	2018	USA	Cons.	40	26 (65.0%)	NR	30.6 ± 7.4; 20.0–50.0	27.5 ± 4.3	NR	NR	24	iHOT at 6 months post‐intervention
			HAS	40	21 (52.5%)	NR	29.7 ± 7.4; 21.0–44.0	28.2 ± 4.4	NR	NR		
Martin et al. [16]	2021	USA	Cons.	44	20 (45.5%)	Cam: 23 (52.3%)	49.1; 47.7–50.6	26.8; 25.6–28.0	NR	NR	NR	HHS at 3 months post‐intervention
						Pincer: 21 (47.7%)						
	2021	USA	HAS	46	23 (50.0%)	Cam: 18 (39.1%)	49.6; 47.7–54.5	27.1; 25.8–28.4	NR	NR		
						Pincer: 24 (52.2%)						
						Mixed: 4 (8.7%)						
Murphy et al. [17]	2023	Australia	Cons.	46	24 (52.2%)	Cam: 31 (67.4%)	32.7 ± 9.0	24.2 ± 2.5	1043 ± 946	70.7 ± 16.1	NR	iHOT at 12 months post‐intervention
						Pincer: 7 (15.2%)						
						Mixed: 8 (17.4%)						
			HAS	45	27 (60.0%)	Cam: 27 (60.0%)	33.8 ± 11.9	24.2 ± 3.5	691 ± 551	70.3 ± 11.6		
						Pincer: 8 (17.8%)						
						Mixed: 10 (22.2%)						
Palmer et al. [18]	2019	UK	Cons.	110	37 (33.6%)	Cam: 104 (94.5%)	36.0 ± 9.9; 18.0–60.0	26.6 ± 4.8; 18.0–41.0	NR	NR	8	HOS‐ADL at 8 months post‐intervention
						Mixed: 6 (5.5%)						
			HAS	112	38 (33.9%)	Cam: 104 (92.9%)	36.4 ± 9.6; 18.0–59.0	25.9 ± 4.8; 17.0–42.0	NR	NR		
						Pincer: 1 (0.9%)						
						Mixed: 7 (6.2%)						
Cons				489	259 (59.1%)	Cam: 340 (75.7%) Pincer: 53 (11.8%) Mixed: 56 (12.5%)	35.8; 30.6–49.1	25.8; 24.1–27.5	936.0; 548.0–1217.0	69.0; 64.0–70.7		
HAS				484	251 (51.9%)	Cam: 322 (72.5%) Pincer: 57 (12.8%) Mixed: 65 (14.6%)	36.1; 29.7–49.6	25.8; 23.5–28.2	848.7; 691.0–1125.0	67.8; 61.0–70.3		
Total				973	510 (52.4%)	Cam: 662 (74.1%) Pincer: 110 (12.3%) Mixed: 121 (13.5%)	37.2; 29.7–49.6	25.8; 23.5–28.2	892.3; 548.0–1217.0	68.4; 61.0–70.7	14; 8.0–24.0	

Abbreviations: BMI, body mass index; Cons., conservative; FAIS, femoroacetabular impingement syndrome; HAS, hip arthroscopy; HHS, Harris Hip Score; HOS‐ADL, Hip Outcome Score—Activities of Daily Living; iHOT, International Hip Outcome Tool; MCID, minimal clinically important difference; PROM, patient‐reported outcome measure; SD, standard deviation.

### 3.3 Quality Assessment

**FIGURE 2:**
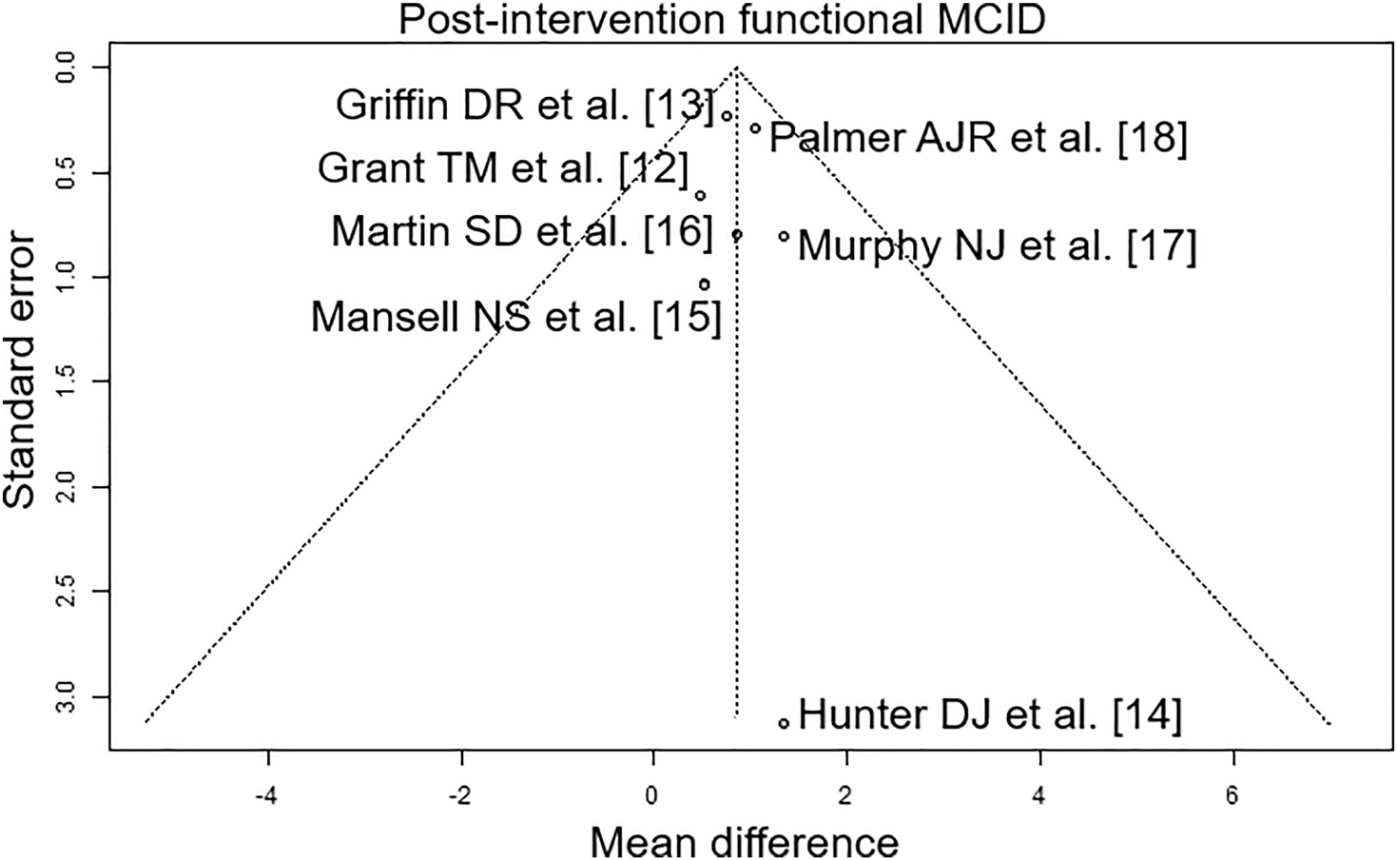
Funnel plot of post‐intervention functional MCID. MCID, minimum clinically important difference.

**FIGURE 3:**
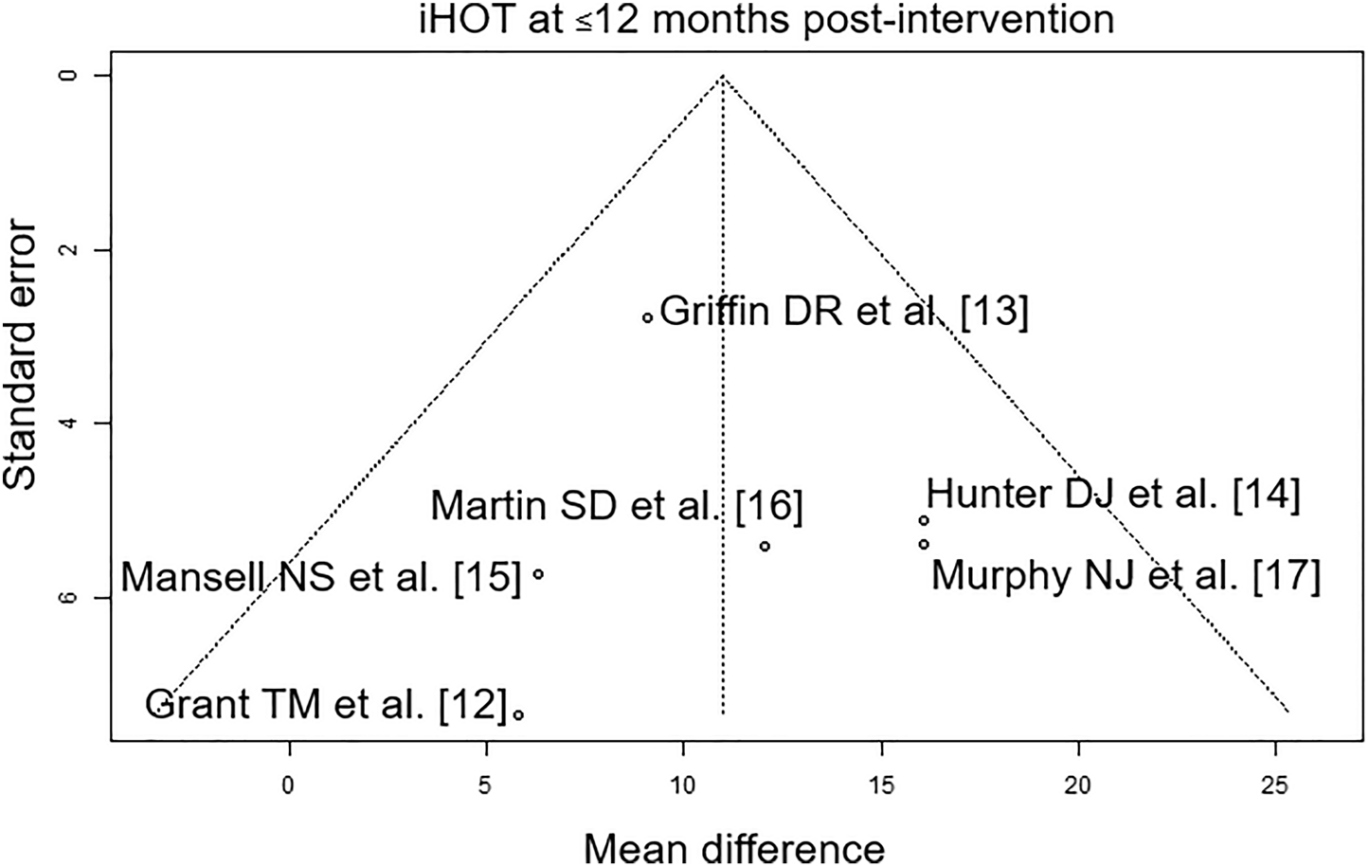
Funnel plot of iHOT at ≤ 12 months post‐intervention. iHOT, International Hip Outcome Tool.

**FIGURE 4:**
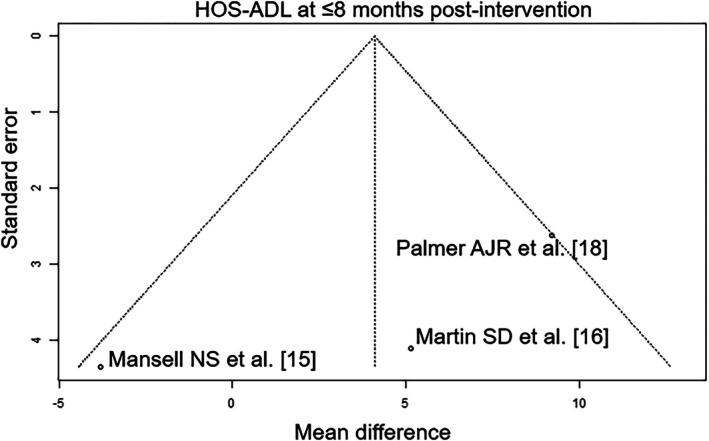
Funnel plot of HOS‐ADL at ≤ 8 months post‐intervention. HOS‐ADL, Hip Outcome Score—Activities of Daily Living.

Of the seven RCTs included, four were rated with a low risk of bias [12, 14, 17, 18], one was rated with a moderate risk of bias [15], and two were rated with a high risk of bias [13, 16] (Table 4). The outcome parameters “post‐intervention functional MCID” and “iHOT at ≤ 12 months post‐intervention” had a high level of evidence and the outcome “HOS‐ADL at ≤ 8 months post‐intervention” parameter had a moderate level of evidence (Table 5). The funnel plots showed the following results in publication bias assessment: low publication bias: post‐intervention functional MCID (Figure 2); iHOT at ≤ 12 months post‐intervention (Figure 3) and HOS‐ADL at ≤ 8 months post‐intervention (Figure 4).

**TABLE 4:** Risk of bias assessment.

RCT	Random sequence generation	Allocation concealment	Blinding	Complete outcome data	Selective reporting	Other sources of bias	Overall risk of bias
Grant et al. [12]	+	+	+	+	+	?	+
Griffin et al. [13]	+	+	−	+	?	+	−
Hunter et al. [14]	+	+	+	+	+	+	+
Mansell et al. [15]	+	+	−	+	+	+	?
Martin et al. [16]	+	+	−	+	+	−	−
Murphy et al. [17]	+	+	+	+	+	+	+
Palmer et al. [18]	+	+	+	+	+	+	+

*Note*: (+), Low risk of bias; (?), some concerns; (−), high risk of bias.

Abbreviation: RCT, randomized controlled trial.

**TABLE 5:** Level of evidence assessment according to GRADE recommendations.

No. of studies	Design	Risk of bias	Inconsistency	Indirectness	Imprecision	Other considerations	Quality of evidence
Post‐intervention functional MCID							
7	RCT	Moderate	No serious inconsistency	No serious indirectness	No serious imprecision	—	High
iHOT at ≤ 12 months post‐intervention							
6	RCT	Moderate	No serious inconsistency	No serious indirectness	No serious imprecision	—	High
HOOS‐ADL at ≤ 8 months post‐intervention							
3	RCT	Moderate	Serious inconsistency	No serious indirectness	No serious imprecision	—	Moderate

Abbreviations: HOS‐ADL, Hip Outcome Score—Activities of Daily Living; iHOT, International Hip Outcome Tool; MCID, minimum clinically important difference; RCT, randomized controlled trial.

### 3.4 Meta‐Analysis

#### 3.4.1 Post‐Intervention Functional MCID

**FIGURE 5:**
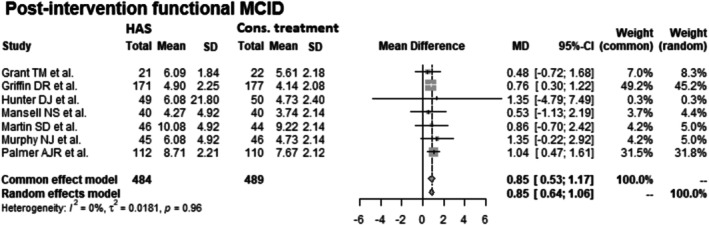
Forest plot of post‐intervention functional MCID. CI, confidence interval; Cons., conservative; HAS, hip arthroscopy; MCID, minimum clinically important difference; MD, mean difference; SD, standard deviation.

Data from 973 patients from 7 RCTs [12, 13, 14, 15, 16, 17, 18] were pooled (Figure 5, Table 6), with the conservative treatment group consisting of 489 patients and the HAS group consisting of 484 patients. The HAS group had a 0.85 statistically significant higher post‐intervention functional MCID than the conservative treatment group (Common effect model: MD: 0.85 CIs 0.53–1.17; Random effects model: MD: 0.85 CIs 0.64–1.06; *I* ^2^ = 0%; *τ* ^2^ = 0.02; *p* = 0.96).

**TABLE 6:** Overview of the most important results of the meta‐analysis.

Outcome parameter	RCTs, *N*	Patients, *N*	Treatment effect	*p*	*I* ^2^	*τ* ^2^	Egger *p*	Begg *p*	References
Post‐intervention functional MCID	7	973	0.85	< 0.01***	0.00	0.02	0.92	0.76	[12, 13, 14, 15, 16, 17, 18]
iHOT at ≤ 12 months post‐intervention	6	751	10.98	< 0.01**	0.00	7.52	0.73	0.45	[12, 13, 14, 15, 16, 17]
HOS‐ADL at ≤ 8 months post‐intervention	3	392	4.10	0.40	0.69	29.88	0.33	0.30	[15, 16, 18]

Abbreviations: HOS‐ADL, Hip Outcome Score—Activities of Daily Living; iHOT, International Hip Outcome Tool; MCID, minimum clinically important difference; RCT, randomized controlled trial, *, significant; **, more significant; ***, highly significant.

#### 3.4.2 iHOT at ≤ 12 Months Post‐Intervention

**FIGURE 6:**
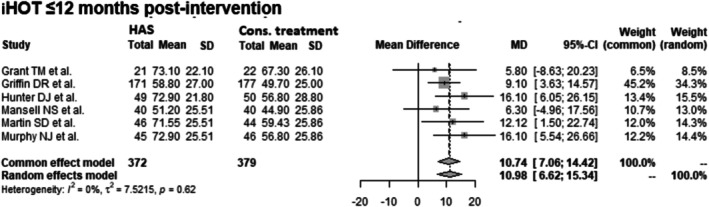
Forest plot of iHOT at ≤ 12 months post‐intervention. CI, confidence interval; Cons., conservative; HAS, hip arthroscopy; iHOT, International Hip Outcome Tool; MD, mean difference; SD, standard deviation.

Data from 751 patients from 6 RCTs [12, 13, 14, 15, 16, 17] were pooled (Figure 6, Table 6), with the conservative treatment group consisting of 379 patients and the HAS group consisting of 372 patients. The HAS group had a 10.74 statistically significant higher iHOT at ≤ 12 months post‐intervention than the conservative treatment group (common effect model: MD: 10.74 CIs 7.06–14.42; random effects model: MD: 10.98 CIs 6.62–15.34; *I* ^2^ = 0%; *τ* ^2^ = 7.52; *p* = 0.62).

#### 3.4.3 HOS‐ADL at ≤ 8 Months Post‐Intervention

**FIGURE 7:**
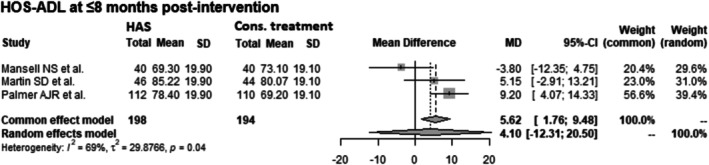
Forest plot of HOS‐ADL at ≤ 8 months post‐intervention. CI, confidence interval; Cons., conservative; HAS, hip arthroscopy; HOS‐ADL, Hip Outcome Score—Activities of Daily Living; MD, mean difference; SD, standard deviation.

Data from 392 patients from 3 RCTs [15, 16, 18] were pooled (Figure 7, Table 6), with the conservative treatment group consisting of 194 patients and the HAS group consisting of 198 patients. HOS‐ADL at ≤ 8 months post‐intervention showed high heterogeneity. In the case of high heterogeneity, we retained the results of the random effects model. There was no difference between the HAS group and the conservative treatment group in HOS‐ADL at ≤ 8 months post‐intervention using a random effects model (Common effect model: MD: 5.62 CIs 1.76–9.48; Random effects model: MD: 4.10 CIs −12.31 to 20.50; *I* ^2^ = 69%; *τ* ^2^ = 29.88; *p* = 0.04).

## 4 Discussion

### 4.1 Summary of Findings

The HAS group had a statistically significant 0.85 higher post‐intervention functional MCID and a statistically significant 10.74 higher iHOT at ≤ 12 months post‐intervention than the conservative treatment group. There was no difference in HOS‐ADL between the HAS and conservative treatment groups at ≤ 8 months post‐intervention.

### 4.2 Comparison With Previous Meta‐Analyses

A total of 11 meta‐analyses [1, 2, 3, 4, 5, 6, 7, 8, 9, 10, 11] have been published between 2020 and 2025 comparing the outcomes of conservatively treated FAIS patients with those treated with HAS. Unfortunately, these meta‐analyses had serious methodological flaws (Tables 1 and 2) and did not report the heterogeneity estimator used. Almost none of these meta‐analyses [1, 2, 3, 4, 5, 6, 7, 8, 10, 11] appears to have used a HK adjustment [44], which, as shown in recent methodological work [36], can lead to misleading or overly narrow confidence intervals. Saueressig et al. performed a critical appraisal [37] of six [1, 2, 3, 4, 5, 10] of these meta‐analyses [1, 2, 3, 4, 5, 6, 7, 8, 9, 10, 11], each of which included the same three primary RCTs [13, 15, 18]. The authors [37] recalculated these meta‐analyses [1, 2, 3, 4, 5, 10] using appropriate statistical methods and found that these meta‐analyses present unreliable results due to methodological flaws [37]. The main reason for conducting this meta‐analysis was to overcome these shortcomings and to try to provide reliable results on the topic examined. Therefore, we used a Sidik‐Jonkman heterogeneity estimator with HK [44] adjustment. We performed a direct head‐to‐head comparison of mean differences (MDs) with 95% confidence intervals between the two treatment groups. We calculated a common effect and random effects model, and followed the results of the random effects model in the case of higher heterogeneity (*I* ^2^ ≥ 50%).

The present conventional frequentist meta‐analysis complements a recently published multilevel meta‐analysis [9] by the same author group. Both analyses were conducted under the same PROSPERO protocol (CRD42024551224), but differ in statistical approach and study inclusion. The previous multilevel meta‐analysis [9] synthesized 21 RCTs [12, 13, 14, 15, 16, 17, 18, 20, 21, 22, 23, 24, 25, 26, 27, 28, 29, 30, 31, 32, 33] with varying designs using indirect subgroup comparisons and a broader evidence base (search until June 30, 2024). In contrast, the present frequentist meta‐analysis focuses on seven directly comparable RCTs [12, 13, 14, 15, 16, 17, 18] (search updated to March 31, 2025) and applies the Sidik‐Jonkman estimator with Hartung‐Knapp adjustment to ensure robust variance estimation in direct two‐arm comparisons of conservative treatment versus HAS.

Although the multilevel model [9] offered valuable broad‐scope synthesis, the current approach provides more targeted insights through direct comparisons, testing the robustness and generalizability of earlier findings using an alternative modeling strategy. Thus, this analysis contributes to both methodological triangulation and enhanced clinical interpretability of treatment effects. Together, the two complementary analyses strengthen the overall evidence base, with both consistently demonstrating a benefit of HAS over conservative treatment in FAIS patients.

Meta‐analyses on conservative treatment versus HAS for FAIS have been instrumental in advancing scientific understanding and guiding clinical decision‐making. By synthesizing data from multiple studies, they provide a higher level of evidence than individual trials, helping to identify treatment effects, assess variability, and highlight gaps in current research. However, the impact of these meta‐analyses depends heavily on methodological rigor. Flaws such as improper handling of heterogeneity and lack of statistical adjustments can lead to misleading conclusions, underscoring the need for continuous refinement of analytical approaches. When conducted with appropriate statistical methods, meta‐analyses serve as a cornerstone of evidence‐based medicine, shaping treatment guidelines and improving patient outcomes.

Another major advantage of this meta‐analysis is that it first calculated the MCID of the functional outcomes reported in the primary studies and then synthesized the result into a single functional outcome parameter. This makes it possible to draw definitive conclusions about the clinical significance of the results. We found that the post‐intervention functional MCID was statistically significant 0.85 higher in the HAS group compared to the conservative treatment group. A third major advantage of the present meta‐analysis is that it included seven RCTs, which is significantly more than previous meta‐analyses [1, 2, 3, 4, 5, 6, 7, 8, 10, 11]. Most of these meta‐analyses [1, 2, 3, 4, 5, 10] included only three primary trials [13, 15, 18], which obviously provides weaker evidence.

### 4.3 Long‐Term Implications and Future Research

A very interesting systematic review by Frank et al. [51] including 26 primary studies with 2114 asymptomatic hips (57.2% men; 42.8% women; mean age of participants was 25.3 ± 1.5 years) found that the prevalence of asymptomatic labral deformity was 37%, ranging from 7% to 100% between the included primary studies. It was 54.8% in athletes compared to 23.1% in the general population. The prevalence of asymptomatic hips with pincer deformity was 67%, ranging from 61% to 76% in the included primary studies. It is well known that asymptomatic FAIS will eventually lead to hip pain and finally to hip osteoarthritis. It should be considered whether orthopedic surgeons should be more generous with the indication of HAS in FAIS, knowing the consequences of untreated FAIS, and try to work towards the prevention of FAIS‐related and FAIS‐caused sequelae.

The evidence in this meta‐analysis is predominantly limited to short‐term follow‐up RCTs, with most outcomes assessed at ≤ 12 months. In the literature, there is one mid‐term follow‐up RCT [50] relevant to the present meta‐analysis. However, this RCT shares the same patient cohort as an already included RCT [18] and was therefore not eligible for inclusion. In this study [50], 112 patients were in the HAS group, while 110 received conservative treatment. At the 38‐month follow‐up, the HAS group demonstrated a mean HOS‐ADL score of 84.2 ± 17.4 points, compared to 74.2 ± 21.9 points in the conservative treatment group, resulting in a significant difference of 8.9 points (95% CI: 7.0–10.8) in favor of HAS. While our meta‐analysis demonstrates that HAS yields a higher likelihood of achieving MCIDs compared to conservative treatment, it remains unclear whether these benefits persist in the long term. Critical questions remain regarding the durability of functional improvements and whether HAS influences the natural history of FAIS, such as delaying the progression to osteoarthritis. To address this gap, future high‐quality studies should focus on mid‐ to long‐term outcomes. Encouraging extended follow‐up in RCTs or leveraging registry data will be essential to strengthen the evidence base and determine whether the early advantages of HAS translate into sustained long‐term benefits.

### 4.4 Strengths and Limitations

This meta‐analysis has some strengths and limitations: (i) a Sidik‐Jonkman heterogeneity estimator with HK adjustment was used; (ii) common effect and random effects models were calculated, with the random effects model used in cases of higher heterogeneity (< 50%); (iii) the inclusion of primary studies was restricted to RCTs; (iv) the MCIDs of the functional outcomes reported in the primary trials were calculated, and the result was then synthesized into a single functional outcome parameter, which yields more clinically relevant and robust conclusions; (v) the general limitations of well‐conducted meta‐analyses apply to this meta‐analysis; (vi) this meta‐analysis included RCTs with a predominantly short‐term follow‐up (≤ 12 months), leaving uncertainty about the long‐term sustainability of HAS's benefits and its potential impact on disease progression.

## 5 Conclusion

This meta‐analysis of 7 RCTs [12, 13, 14, 15, 16, 17, 18] with a total of 973 FAIS patients using high‐quality statistical methods showed a statistically significant higher post‐intervention functional MCID and iHOT at ≤ 12 months post‐intervention in favor of the HAS group compared to the conservative treatment group. No statistically significant differences were observed in HOS‐ADL at ≤ 8 months post‐intervention.

## Author Contributions

**Nikolai Ramadanov:** conceptualization, investigation, writing – original draft, methodology, validation, visualization, writing – review and editing, software, formal analysis, project administration, data curation, supervision, resources. **Jonathan Lettner:** methodology, data curation, writing – review and editing, investigation. **Maximilan Voss:** methodology, data curation, investigation. **Robert Prill:** writing – review and editing, supervision. **Robert Hable:** conceptualization, investigation, methodology, software, formal analysis, validation, visualization, writing – review and editing, supervision. **Dobromir Dimitrov:** supervision, writing – review and editing. **Roland Becker:** writing – review and editing, supervision.

## Conflicts of Interest

The authors declare no conflicts of interest.

## Supporting information


**Data S1.** Supporting Information.

**Data S2.** Supporting Information.

## Data Availability

Raw data extraction sheet is available in supplement.
